# *Knockdown* do RNA Longo Não Codificante ZEB1-AS1 Acelera a Hipertrofia Cardíaca pela Via miR-186-5p/HDAC2

**DOI:** 10.36660/abc.20240703

**Published:** 2025-12-18

**Authors:** Bingfeng Cao, Qingxia Liu, Xiaoyuan Zhang, Yufeng Wang

**Affiliations:** 1 Department of Cardiology Weihai Central Hospital Shandong China Department of Cardiology – Weihai Central Hospital, Shandong – China; 2 Department of Outpatient Weihai Central Hospital Shandong China Department of Outpatient – Weihai Central Hospital, Shandong – China

**Keywords:** Cardiomegalia, RNA Longo não Codificante, Histona Desacetilase 2

## Abstract

**Fundamento:**

O papel do RNA longo não codificante ZEB1-AS1 na hipertrofia cardíaca (HC) permanece incerto.

**Objetivos:**

Investigar a função do ZEB1-AS1 no desenvolvimento e na progressão da HC, bem como elucidar seu mecanismo molecular subjacente.

**Métodos:**

Os níveis de expressão de RNA foram quantificados por PCR quantitativa em tempo real. A área de superfície das células AC16 foi avaliada por coloração por imunofluorescência. A expressão proteica foi analisada por *Western blotting*. As interações entre RNAs foram examinadas por ensaios com repórter de luciferase e por imunoprecipitação de RNA. A significância estatística foi definida como p < 0,05.

**Resultados:**

A expressão de ZEB1-AS1 esteve aumentada em tecidos miocárdicos e em células AC16 estimuladas com isoproterenol (ISO). O *knockdown* de ZEB1-AS1 atenuou as respostas hipertróficas induzidas por ISO. Mecanisticamente, ZEB1-AS1 modulou a expressão de desacetilase de histonas 2 (HDAC2) atuando como “esponja molecular” para miR-186-5p. De forma consistente, o *knockdown* de ZEB1-AS1 reduziu a HDAC2 e diminuiu a expressão de marcadores hipertróficos, incluindo o peptídeo natriurético tipo B, o peptídeo natriurético atrial e a cadeia pesada de miosina β, restringindo assim a progressão da HC.

**Conclusões:**

ZEB1-AS1 encontra-se superexpresso em tecidos miocárdicos e em células AC16 estimuladas com ISO. Nossos achados indicam que o eixo ZEB1-AS1/miR-186-5p/HDAC2 contribui para a HC, oferecendo uma base mecanística e um potencial alvo terapêutico para intervenção clínica.

## Graphical abstract

**Figura Central:**
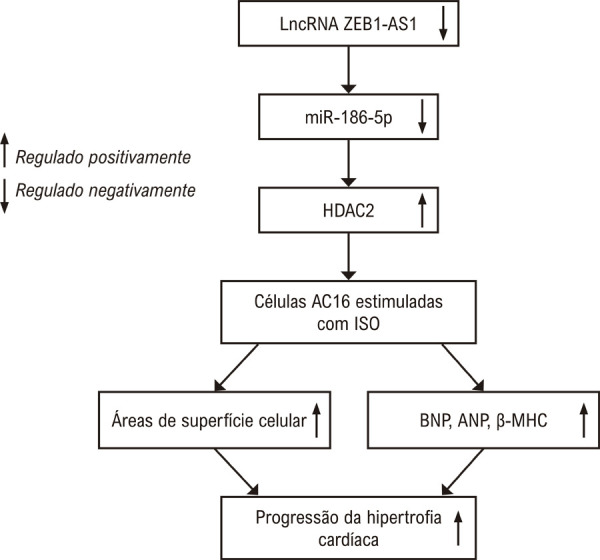
*Knockdown* do RNA Longo Não Codificante ZEB1-AS1 Acelera a Hipertrofia Cardíaca pela Via miR-186-5p/HDAC2

Knockdown do RNA Longo Não Codificante ZEB1-AS1 Acelera a Hipertrofia Cardíaca Pela Via miR-186-5p/HDAC2. ANP: peptídeo natriurético atrial; BNP: peptídeo natriurético tipo B; β-MHC: cadeia pesada de miosina β; HDAC2: desacetilase de histonas 2; ISSO: isoproterenol; LncRNA: RNA longo não codificante.

## Introdução

A hipertrofia cardíaca (HC) caracteriza-se pelo aumento do tamanho dos cardiomiócitos, e não pelo aumento de seu número. Trata-se, principalmente, de uma resposta compensatória ao estresse biomecânico, destinada a preservar o débito cardíaco e manter a homeostase fisiológica.^1,2^ A HC é amplamente classificada como patológica ou fisiológica. A HC patológica associa-se comumente à apoptose de cardiomiócitos, fibrose miocárdica, arquitetura celular desorganizada, alterações epigenéticas e reativação aberrante de programas gênicos fetais, incluindo os marcadores peptídeo natriurético tipo B (BNP), peptídeo natriurético atrial (ANP) e cadeia pesada de miosina β (β-MHC).^3-5^ A persistência da HC patológica pode levar a desfechos clínicos graves, como arritmia, remodelamento cardíaco maladaptativo, insuficiência cardíaca (IC), infarto do miocárdio e morte súbita cardíaca.^6,7^ Apesar dos avanços terapêuticos, os desfechos — particularmente na IC relacionada à HC — permanecem subótimos, com cerca de 50% de sobrevida em 5 anos após o diagnóstico.^8^ Elucidar os mecanismos moleculares que impulsionam o desenvolvimento e a progressão da HC é, portanto, uma prioridade de pesquisa.

Os RNAs não codificantes (ncRNAs) — incluindo RNAs circulares (circRNAs), RNAs longos não codificantes (lncRNAs) e micro RNAs (miRNAs) — têm atraído considerável atenção por seus papéis em diversos processos regulatórios, principalmente por meio da modulação da expressão de RNAs codificadores de proteína.^9-13^ Os lncRNAs, definidos como transcritos não codificadores com ≥ 200 nucleotídeos e sem capacidade de codificação proteica, emergiram como reguladores-chave em um amplo espectro de condições patológicas.^14,15^ Numerosos estudos mostram que lncRNAs contribuem para a patogênese de doenças como nefropatia diabética,^16^ hipertensão pulmonar,^17^ neoplasias malignas^18^ e doenças cardiovasculares.^19-21^ Evidências crescentes também implicam lncRNAs no desenvolvimento e na progressão da HC. Por exemplo, o silenciamento do lncRNA TUG1 atenua a HC ao modular a via miR-497/MEF2C,^22^ o ncRNA PVT1 promove a HC patológica atuando como esponja molecular para miR-196b^23^ e o lncRNA NEAT1 acelera a HC por meio do eixo miR-19a-3p/SMYD2.^24^ O RNA longo não codificante ZEB1-AS1 (lncRNA ZEB1-AS1), um lncRNA recentemente identificado e inicialmente estudado em câncer, tem sido implicado em múltiplos processos patológicos;^25^ contudo, seu papel na HC permanece amplamente indefinido.

Os miRNAs constituem uma classe de ncRNAs curtos e altamente conservados (tipicamente > 22 nucleotídeos [nt]) que reprimem a expressão de mRNAs-alvo.^26^ O miR-186-5p é um miRNA bem caracterizado envolvido na regulação de várias doenças humanas, incluindo condições cardiovasculares.^27-29^ Entretanto, seu papel específico na HC ainda não é bem compreendido. A desacetilase de histonas 2 (HDAC2), membro da família de desacetilases de histonas, tem sido implicada em diversos estados patológicos, incluindo HC.^30-32^ Resta determinar se o eixo regulatório miR-186-5p/HDAC2 participa da progressão da HC mediada por ZEB1-AS1.

Este estudo teve como objetivo investigar o papel do ZEB1-AS1 no desenvolvimento e na progressão da HC e delinear seu mecanismo subjacente, a fim de identificar um potencial alvo terapêutico.

## Métodos

### Espécimes de tecido cardíaco humano

Coletamos amostras de tecido cardíaco de 20 pacientes com hipertrofia ventricular esquerda (HVE) — secções de coração obtidas durante transplante cardíaco — e de 10 corações de doadores sem histórico de doença cardíaca, em nosso hospital, entre março de 2018 e setembro de 2023. A Tabela 1 resume as características clínicas de pacientes e doadores.

**Tabela 1:** – Sequências de *primers* utilizadas no qRT-PCR

Gene	*Forward* (5′ → 3′)	*Reverse* (5′ → 3′)
ZEB1-AS1	5′-CCGTGGGCACTGCTGAAT-3′	5′-CTGCTGGCAAGCGGAACT-3′
miR-186-5p	5′-CAAAGAAUUCUCCUUUUGGGCU-3′	5′-CCCAAAAGGAGAAUUCUUUGUU-3′
HDAC2	5′-TGAGATTCCCAATGAGTTGCCA-3′	5′-TACTGACATCTGGTCAGACA-3′
ANP	5′-CAACGCAGA-CCTGATGGATTT-3′	5′-AGCCCCCGC-TTCTTCATTC-3′
BNP	5′-TGGAAACGTCCGGGTTACAG-3′	5′-CTGATCCGGTCCATCTTCCT-3′
β-MHC	5′-CCGAGTCCCAGGTCAACAA-3′	5′-CTTCACGGGCACCCTTGGA-3′
U6	5′-GCTTCGGCAGCACATATACTAAAAT-3′	5′-CGCTTCACGAATTTGCGTGTCAT-3′
GAPDH	5′-CCATCAACGACCCCTTCATT-3′	5′-GACCAGCTTCCCATTCTCAG-3′

ANP: peptídeo natriurético atrial; BNP: peptídeo natriurético tipo B; GAPDH: gliceraldeído-3-fosfato desidrogenase; HDAC2: histone deacetylase 2; miR-186-5p: microRNA-186-5p; qRT-PCR: reação em cadeia da polimerase quantitativa em tempo real; U6: RNA nuclear pequeno U6; ZEB1-AS1: zinc finger E-box binding homeobox 1 antisense RNA 1; β-MHC: cadeia pesada de miosina β.

Os critérios de inclusão para HVE foram espessura do septo interventricular em diástole e/ou espessura da parede posterior do VE ≥ 1,2 cm, com fração de ejeção do VE > 40% à ecocardiografia. Critérios de exclusão incluíram IC sistólica, neoplasia, infecção aguda, BNP > 400 ng/L, doença de Parkinson, fibrose pulmonar idiopática, choque, cirrose hepática ou tratamento recente com interferon ou fenobarbital.^33^ Os corações de doadores eram provenientes de indivíduos falecidos por causas não cardíacas; os tecidos foram coletados entre 0,5 e 6 h pós-morte. Todas as amostras de tecido cardíaco foram imediatamente congeladas por *snap-freezing* em nitrogênio líquido (Delun, Xangai, China) antes da extração de RNA.

O estudo foi aprovado pelo Comitê de Ética Institucional de nosso hospital, e todos os participantes forneceram consentimento informado. Os procedimentos de pesquisa aderiram aos princípios da revisão de 2024 da Declaração de Helsinque da Associação Médica Mundial.^34^

### Cultura celular e transfecção

A linhagem de cardiomiócitos humanos AC16 foi obtida da American Type Culture Collection (EUA). As células foram cultivadas em *Dulbecco’s Modified Eagle Medium* (DMEM; HyClone Laboratories, EUA) suplementado com 20% de soro fetal bovino (Gibco, EUA) e mantidas em incubadora umidificada a 37 °C com 5% de CO_2_. Para estabelecer um modelo in vitro de HC, células AC16 foram tratadas com 10 µmol/L de isoproterenol (ISO; Sigma-Aldrich, EUA), conforme protocolos publicados.^35^

Os construtos *short hairpin RNA–negative control* (sh-NC) e sh-ZEB1-AS1 foram obtidos da GeneChem (Xangai, China). *Mimics* de miR-186-5p, um inibidor de miR-186-5p e seus respectivos controles negativos foram adquiridos da RiboBio (Guangzhou, China). As transfecções celulares foram realizadas com Lipofectamine 3000 (Sigma-Aldrich, EUA), seguindo as instruções do fabricante.

### PCR quantitativa em tempo real

O RNA total foi extraído de amostras de tecido cardíaco humano e de células AC16 usando o reagente TRIzol (Sigma-Aldrich, EUA). A síntese de cDNA e a amplificação foram realizadas com SYBR Green Master Mix (Takara, Japão), conforme instruções do fabricante. GAPDH e U6 serviram como controles endógenos para normalização da expressão de lncRNA/mRNA e miRNA, respectivamente. A expressão relativa foi calculada pelo método 2^Ct^. A Tabela 2 apresenta as sequências de primers utilizadas no qRT-PCR.

**Tabela 2:** – Características clínicas de doadores saudáveis e pacientes com HVE

Variável	Saudáveis (n = 10)	HVE (n = 20)	Valor p
Idade (anos)	67 ± 8	68 ± 12	0,8141
Masculino/feminino (n/n)	4/6	11/9	0,4390
Tabagismo, n (%)	4 (40%)	11 (55%)	0,4390
DM, n (%)	3 (30%)	12 (60%)	0,1210
Hipertensão, n (%)	4 (40%)	18 (90%)	0,0040
SCA, n (%)	2 (20%)	14 (70%)	0,0100
FA, n (%)	0 (0%)	5 (25%)	0,0830
Glicose em jejum (mmol/L)	5,51 ± 0,54	6,23 ± 1,85	0,2417
PAS (mmHg)	115,25 ± 18,25	159,91 ± 28,24	0,0001
PAD (mmHg)	73,52 ± 12,25	95,41 ± 15,36	0,0005
HDL-C (mmol/L)	1,36 ± 0,11	1,15 ± 0,29	0,0365
LDL-C (mmol/L)	2,61 ± 0,56	3,05 ± 0,81	0,1354
BNP (ng/L)	129,61 ± 74,12	158,36 ± 95,21	0,4112
FE (%)	78,55 ± 5,81	58,25 ± 6,25	0,0001
Diâmetro do VE (cm)	4,25 ± 0,45	4,38 ± 0,66	0,5807
DDFVE (cm)	4,55 ± 0,50	4,99 ± 0,91	0,1673
EIVd (cm)	0,98 ± 0,08	1,24 ± 0,13	0,0001
EPPVEd (cm)	0,95 ± 0,12	1,29 ± 0,12	0,0001
ZEB1-AS1 relativo (média ± DP)	0,895 ± 0,394	2,194 ± 0,633	0,0001

BNP: peptídeo natriurético tipo B; DDFVE: diâmetro diastólico final do ventrículo esquerdo; DM: diabetes melito; EIVd: espessura do septo interventricular em diástole; EPPVEd: espessura da parede posterior do ventrículo esquerdo em diástole; FA: fibrilação atrial; FEVE: fração de ejeção do ventrículo esquerdo; HDL-C: colesterol de lipoproteína de alta densidade; HVE: hipertrofia do ventrículo esquerdo; LDL-C: colesterol de lipoproteína de baixa densidade; PAD: pressão arterial diastólica; PAS: pressão arterial sistólica; SCA: síndrome coronariana aguda.

### Western blotting

A extração de proteínas de células AC16 foi realizada conforme descrito anteriormente.^36^ Em resumo, as células foram lisadas em tampão *radioimmunoprecipitation assay* e as proteínas foram desnaturadas por fervura. Quantidades iguais de proteína foram separadas por eletroforese em gel de poliacrilamida com dodecil sulfato de sódio a 10% (SDS-PAGE) e transferidas para membranas de polifluoreto de vinilideno (PVDF; Millipore, EUA). As membranas foram bloqueadas com 1% de albumina sérica bovina (BSA) por 1 h à temperatura ambiente e lavadas com solução salina tamponada com fosfato (PBS). As *blots* foram incubadas a 4 °C, durante a noite, com anticorpos primários contra ANP (1:2000, Abcam, EUA), BNP (1:2000, CST, EUA), β-MHC (1:2000, CST, EUA) e GAPDH (1:2000, Abcam, EUA). Após lavagem, as membranas foram incubadas com anticorpo secundário (1:10.000, Jackson, EUA) por 2 h à temperatura ambiente. As bandas foram visualizadas por quimioluminescência aprimorada (Amersham, Reino Unido) e quantificadas no ImageJ (National Institutes of Health, EUA).

### Coloração por imunofluorescência

Células AC16 foram fixadas com paraformaldeído a 4% a 37 °C por 30 min, lavadas três vezes com PBS e permeabilizadas com Triton X-100 a 0,1% por 15 min. As células foram bloqueadas por 30 min em soro normal de cabra a 10% com 1% de BSA (Sigma-Aldrich) e incubadas, no escuro, durante a noite a 4 °C, com anticorpo primário anti-β-actina (Abcam, EUA) diluído no tampão de bloqueio. Após três lavagens em PBS, as células foram incubadas com o anticorpo secundário apropriado por 2 h a 37 °C, contracoradas com DAPI e imageadas em microscópio de fluorescência (Carl Zeiss). A área de superfície celular foi quantificada no ImageJ.

### Ensaio repórter de dupla luciferase

Sequências de ligação para miR-186-5p em ZEB1-AS1 e na 3′-RNT de HDAC2 foram clonadas em vetores pGL3-Basic (LMAI Bio, Xangai, China). Células AC16 foram cotransfectadas com *mimic* controle negativo ou *mimic* de miR-186-5p juntamente com construções repórter *wild type* (WT) ou mutantes (MUT). Após a transfecção, as células foram coletadas e a atividade de luciferase foi medida com o *Dual-Luciferase Reporter Assay System* (Promega, EUA).

### Imunoprecipitação de RNA

A imunoprecipitação de RNA (RIP) foi realizada com o *RNA-Binding Protein Immunoprecipitation Kit* (Millipore, EUA) para avaliar as interações entre ZEB1-AS1, miR-186-5p e HDAC2, conforme instruções do fabricante. Em resumo, as células foram lisadas em tampão de lise RIP e incubadas com esferas magnéticas conjugadas a anti-Argonaute-2 (Ago2; Abcam, EUA) ou IgG de coelho controle (Abcam, EUA). Após três lavagens com PBS, o RNA coprecipitado com Ago2 ou IgG foi purificado e analisado por qRT-PCR.

### Análise dos dados

As análises foram realizadas no GraphPad Prism versão 7.0.0 para Windows, GraphPad Software, Boston, Massachusetts, EUA; www.graphpad.com. Os dados são apresentados como média ± desvio-padrão (DP). Comparações entre dois grupos utilizaram teste *t* de Student bicaudal e não pareado; diferenças entre múltiplos grupos foram avaliadas por ANOVA de um fator com pós-teste de Bonferroni. As associações entre a expressão de ZEB1-AS1 e variáveis clínicas foram avaliadas com o teste do qui-quadrado. Normalidade e homocedasticidade foram verificadas pelos testes de Shapiro–Wilk e de Levene, respectivamente; p > 0,05 indicou que os pressupostos foram atendidos. A significância estatística foi definida como p < 0,05.

## Resultados

### ZEB1-AS1 em pacientes com HVE e em HC induzida por ISO em células AC16

**Figura 1:**
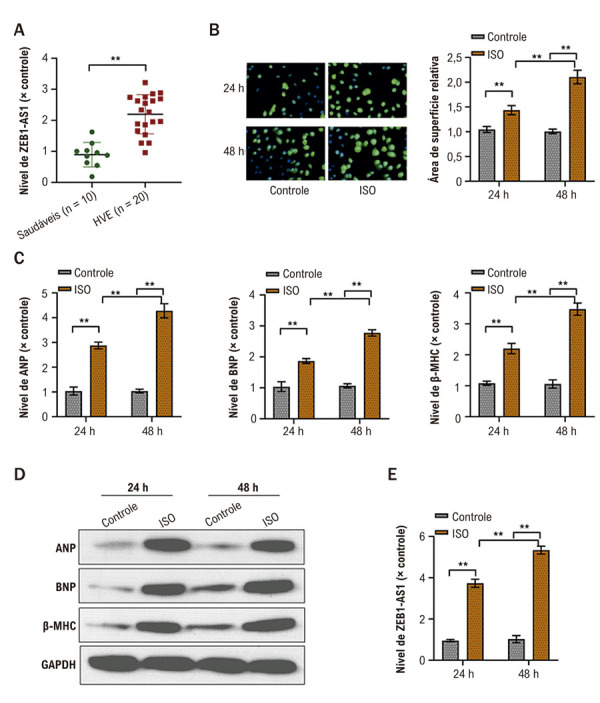
– Superexpressão de ZEB1-AS1 em tecidos com HVE e em células AC16 estimuladas com ISO. A) Expressão de ZEB1-AS1 em corações com HVE (n = 20) versus doadores normais (n = 10) por qRT-PCR; B) Área de superfície de células AC16 após tratamento com PBS ou ISO, avaliada por coloração por imunofluorescência; C) Níveis de mRNA de BNP, ANP e β-MHC em células AC16 após PBS ou ISO por qRT-PCR; D) Níveis de proteína de BNP, ANP e β-MHC em células AC16 após PBS ou ISO por Western blot; E) Expressão de ZEB1-AS1 em células AC16 após PBS ou ISO por qRT-PCR. Todos os experimentos foram realizados em triplicata. *p < 0,05, *p < 0,01. ANP: peptídeo natriurético atrial; BNP: peptídeo natriurético tipo B; β-MHC: cadeia pesada de miosina β; HC: hipertrofia cardíaca; HVE: hipertrofia ventricular esquerda; ISO: isoproterenol; PBS: solução salina tamponada com fosfato; qRT-PCR: reação em cadeia da polimerase quantitativa em tempo real.

Quantificamos inicialmente ZEB1-AS1 em tecidos cardíacos de 20 pacientes com hipertrofia ventricular esquerda (HVE) e de 10 corações de doadores normais por qRT-PCR. ZEB1-AS1 esteve significativamente superexpresso nos tecidos com HVE em comparação aos controles (Figura 1A).

Para modelar a HC in vitro, células AC16 foram tratadas com ISO (10 µM). O ISO aumentou a área de superfície celular às 24 h, atingindo o máximo em 48 h em relação aos controles não tratados (Figura 1B). Em paralelo, o ISO promoveu aumentos dependentes do tempo nos níveis de mRNA e proteína de marcadores hipertróficos — BNP, ANP e β-MHC — quando comparados aos controles (Figuras 1C e 1D), confirmando o estabelecimento bem-sucedido do modelo de HC.

Notavelmente, a expressão de ZEB1-AS1 também aumentou de forma significativa nas células AC16 tratadas com ISO em relação aos controles (Figura 1E), sugerindo um papel de ZEB1-AS1 na progressão da HC.

### A deficiência de ZEB1-AS1 atenua a HC induzida por ISO em células AC16

**Figura 2:**
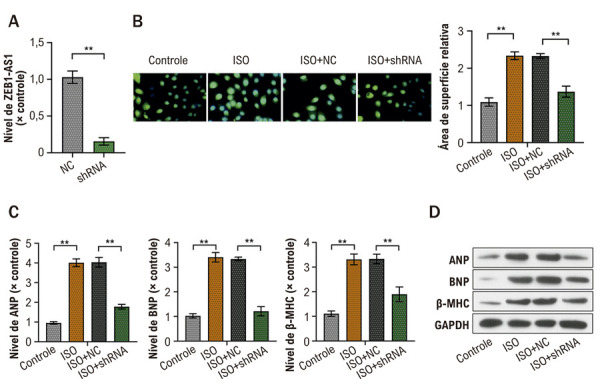
– A deficiência de ZEB1-AS1 atenua a HC induzida por ISO em células AC16. A) Expressão de ZEB1-AS1 em células AC16 transfectadas com sh-NC ou sh-ZEB1-AS1 por qRT-PCR; B) área de superfície das AC16 após estimulação com ISO com sh-NC ou sh-ZEB1-AS1, avaliada por coloração por imunofluorescência; C) níveis de mRNA de BNP, ANP e β-MHC em AC16 estimuladas com ISO com sh-NC ou sh-ZEB1-AS1 por qRT-PCR; D) níveis de proteína de BNP, ANP e β-MHC em AC16 estimuladas com ISO com sh-NC ou sh-ZEB1-AS1 por Western blot. Todos os experimentos foram realizados em triplicata. *p < 0,05, *p < 0,01. ANP: peptídeo natriurético atrial; BNP: peptídeo natriurético tipo B; β-MHC: cadeia pesada de miosina β; HC: hipertrofia cardíaca; ISO: isoproterenol; qRT-PCR: reação em cadeia da polimerase quantitativa em tempo real; sh-NC: short hairpin RNA–negative control; sh-ZEB1-AS1: shRNA direcionado a ZEB1-AS1.

Para avaliar o papel de ZEB1-AS1 na hipertrofia induzida por ISO, células AC16 foram transfectadas com sh-ZEB1-AS1 ou sh-NC. A expressão de ZEB1-AS1 foi significativamente reduzida nas células sh-ZEB1-AS1 em comparação com sh-NC, confirmando *knockdown* eficiente (Figura 2A). O tratamento com ISO aumentou acentuadamente a área de superfície das AC16, consistente com alterações hipertróficas, ao passo que esse aumento foi significativamente atenuado nas células transfectadas com sh-ZEB1-AS1 (Figura 2B). De modo semelhante, o ISO superexpressou os níveis de mRNA e proteína dos marcadores hipertróficos (BNP, ANP e β-MHC); essa indução foi substancialmente atenuada pelo *knockdown* de ZEB1-AS1 (Figuras 2C, D). Esses dados indicam que a redução de ZEB1-AS1 regula negativamente a HC induzida por ISO.

### ZEB1-AS1 regula a expressão de HDAC2 por sequestro de miR-186-5p

**Figura 3:**
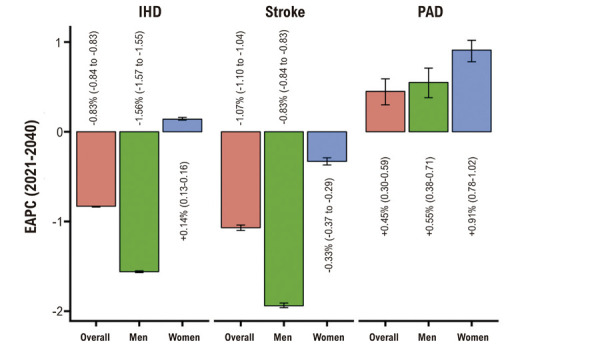
– ZEB1-AS1 regula HDAC2 ao sequestrar miR-186-5p. A) Sítio previsto de ligação de miR-186-5p em ZEB1-AS1 (WT) e construto mutante (MUT) correspondente. B) Ensaio de dupla luciferase mostrando redução da atividade do repórter para ZEB1-AS1-WT, mas não para ZEB1-AS1-MUT, na presença do mimic de miR-186-5p. C) Sítio previsto de ligação de miR-186-5p na 3′-RNT de HDAC2 (WT) e construto MUT. D) Ensaio de dupla luciferase mostrando diminuição da atividade para HDAC2-WT, mas não para HDAC2-MUT, com mimic de miR-186-5p. E) RIP demonstrando enriquecimento de ZEB1-AS1, miR-186-5p e HDAC2 em imunoprecipitados com Ago2. F-G) Níveis de miR-186-5p e de HDAC2 em corações com HVE (n = 20) versus doadores normais (n = 10) por qRT-PCR. Todos os experimentos foram realizados em triplicata. *p < 0,05, *p < 0,01. Ago2: Argonauta-2; CMV: citomegalovírus; HDAC2: desacetilase de histonas 2; LVH: hipertrofia ventricular esquerda; miR-NC: mimic de controle negativo; MUT: mutante; qRT-PCR: reação em cadeia da polimerase quantitativa em tempo real; RIP: imunoprecipitação de RNA; RNT: região não traduzida; WT: wild type.

Utilizamos starBase 2.0 para predizer miRNAs que interagem com ZEB1-AS1 e identificamos o miR-186-5p como candidato (Figura 3A). Um ensaio repórter de dupla luciferase mostrou que a cotransfecção do *mimic* de miR-186-5p com o construto *wild type* de ZEB1-AS1 (ZEB1-AS1-WT) reduziu significativamente a atividade de luciferase em células AC16, ao passo que o construto mutante (ZEB1-AS1-MUT) não teve efeito (Figura 3B), confirmando ligação específica. Usando TargetScan, identificamos em seguida a HDAC2 como alvo a jusante putativo de miR-186-5p (Figura 3C). Essa interação foi validada por ensaio de luciferase: o *mimic* de miR-186-5p diminuiu a atividade do repórter HDAC2-WT, mas não do HDAC2-MUT (Figura 3D). O RIP demonstrou ainda enriquecimento de ZEB1-AS1, miR-186-5p e HDAC2 em imunoprecipitados com Ago2 a partir de lisados de AC16 (Figura 3E), indicando coassociação no complexo de silenciamento induzido por RNA.

Em tecidos miocárdicos de 20 pacientes com HVE versus 10 corações de doadores normais, a qRT-PCR revelou menor miR-186-5p e maior expressão de HDAC2 (Figuras 3F,G). Em conjunto, esses dados sustentam um eixo ZEB1-AS1/miR-186-5p/HDAC2 na HC, pelo qual ZEB1-AS1 modula HDAC2 ao “esponjar” miR-186-5p.

### ZEB1-AS1 promove a HC regulando a via miR-186-5p/HDAC2

**Figura 4:**
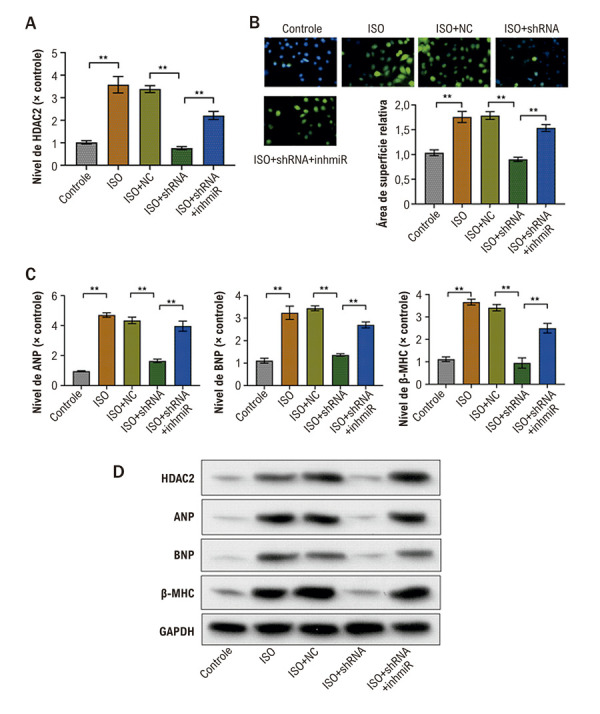
– ZEB1-AS1 promove a HC ao regular a via miR-186-5p/HDAC2. A) mRNA de HDAC2 em células AC16 estimuladas com ISO e transfectadas com os construtos indicados, medido por qRT-PCR; B) área de superfície das AC16 após estimulação com ISO e transfecção com os construtos indicados, avaliada por coloração por imunofluorescência; C) níveis de mRNA de BNP, ANP e β-MHC em AC16 estimuladas com ISO após transfecção, medidos por qRT-PCR; D) níveis de proteína de BNP, ANP e β-MHC em AC16 estimuladas com ISO após transfecção, medidos por Western blot. Todos os experimentos foram realizados em triplicata. *p < 0,05, *p < 0,01. ANP: peptídeo natriurético atrial; BNP: peptídeo natriurético tipo B; β-MHC: cadeia pesada de miosina β; HC: hipertrofia cardíaca; HDAC2: histone deacetylase 2; ISO: isoproterenol; qRT-PCR: reação em cadeia da polimerase quantitativa em tempo real; sh-NC: short hairpin RNA–negative control; sh-ZEB1-AS1: zinc finger E-box binding homeobox 1 antisense RNA 1 direcionado ao short hairpin RNA.

Para testar se ZEB1-AS1 impulsiona a HC via o eixo miR-186-5p/HDAC2, realizamos experimentos de *rescue*. O mRNA de HDAC2 foi medido em células AC16 estimuladas com ISO e transfectadas com sh-NC, sh-ZEB1-AS1 ou cotransfectadas com sh-ZEB1-AS1 mais um inibidor de miR-186-5p. O *knockdown* de ZEB1-AS1 reduziu significativamente o mRNA de HDAC2, e essa redução foi revertida pela inibição de miR-186-5p (Figura 4A). Do mesmo modo, o aumento induzido por ISO na área de superfície celular foi atenuado por sh-ZEB1-AS1, enquanto a inibição concomitante de miR-186-5p aboliu esse efeito (Figura 4B). Em paralelo, o *knockdown* de ZEB1-AS1 diminuiu a expressão de mRNA e proteína dos marcadores hipertróficos induzida por ISO (BNP, ANP e β-MHC), e esses efeitos supressores foram revertidos pela inibição de miR-186-5p (Figuras 4C e 4D). Coletivamente, os resultados indicam que ZEB1-AS1 promove a HC ao regular a via de sinalização miR-186-5p/HDAC2.

## Discussão

Neste estudo, mostramos que ZEB1-AS1 está significativamente superexpresso na HC e que seu silenciamento atenua as respostas hipertróficas induzidas por ISO em células AC16. Mecanisticamente, ensaios de dupla luciferase e RIP confirmaram que miR-186-5p é alvo direto de ZEB1-AS1 e que HDAC2 é alvo de miR-186-5p, estabelecendo um eixo regulatório que contribui para a progressão da HC.

Os lncRNAs foram outrora considerados subprodutos transcripcionais não funcionais,^37^ mas evidências acumuladas indicam que regulam diversos processos biológicos por meio de interações com miRNAs, fatores de transcrição, DNA genômico e proteínas ligantes de RNA. Os lncRNAs têm sido cada vez mais reconhecidos no desenvolvimento cardíaco e na patogênese das doenças cardiovasculares. Por exemplo, o lncRNA MIR217HG exacerba a HC induzida por sobrecarga de pressão via a via miR-138/THBS1,^38^ ADAMTS9-AS1/miR-185-5p/KAT7 atua como uma rede supressora de ceRNA na cardiomiopatia hipertrófica obstrutiva^39^ e o *knockdown* de DANCR mitiga a IC ao atenuar HC e fibrose por meio do eixo miR-758-3p/PRG4/Smad.^35^ ZEB1-AS1, inicialmente caracterizado em câncer, também tem sido implicado em diversas neoplasias,^25,40,41^ mas seu papel na HC permanecia incerto. Aqui, identificamos ZEB1-AS1 como um lncRNA pró-hipertrófico: ele se encontra aumentado em tecidos com HVE e em células AC16 tratadas com ISO, e seu *knockdown* reduz a hipertrofia induzida por ISO, indicando um papel patogênico no desenvolvimento e na progressão da HC.

Os lncRNAs frequentemente atuam em redes de ceRNA para regular a expressão gênica no pós-transcricional, sequestrando miRNAs e, assim, modulando sua disponibilidade para mRNAs-alvo.^42,43^ A importância dos circuitos de ceRNA na HC foi corroborada por diversos estudos.^44-46^ De modo consistente, mostramos que ZEB1-AS1 funciona como sponge para miR-186-5p — um miRNA relatado como reduzido no tecido cardíaco em cardiomiopatia diabética^47^ — aliviando a repressão de HDAC2 e promovendo a hipertrofia induzida por ISO. Ao nosso conhecimento, este é o primeiro relato que implica miR-186-5p na HC, ressaltando seu potencial como ponto de entrada terapêutico.

As HDACs são contribuintes-chave para a patologia cardiovascular.^30-32,48-50^ Trabalhos prévios mostraram que a inibição de HDAC atenua a HC induzida por sobrecarga de pressão ou por infusão de ISO,^51,52^ em linha com nosso modelo in vitro baseado em ISO. Entre as HDACs, a HDAC2 tem recebido atenção particular; a fosforilação na serina 394 é um evento regulatório crítico na progressão da HC.^53^ Por exemplo, o efeito anti-hipertrófico de plantamajoside na HC induzida por ISO associa-se estreitamente à HDAC2,^32^ e EYA4-au1 promove hipertrofia de cardiomiócitos ao recrutar Med11 para superexpressar EYA4, levando à redução de p27^Kip^^1^ e à ativação do eixo CK2α/HDAC2.^54^ Apesar do papel estabelecido da HDAC2, sua regulação a montante via redes lncRNA–miRNA–mRNA tem sido menos clara. Nossos dados indicam que HDAC2 é alvo direto de miR-186-5p e que ZEB1-AS1 promove HC modulando o eixo miR-186-5p/HDAC2.

### Limitações do estudo

Este estudo apresenta algumas limitações. Como lncRNAs frequentemente interagem com múltiplos miRNAs e miRNAs individuais tipicamente regulam numerosos alvos, ZEB1-AS1 pode influenciar vias adicionais relevantes para a HC que não foram examinadas aqui. Esses mecanismos alternativos merecem investigação futura. Além disso, embora o intervalo pós-morte para tecidos de autópsia tenha sido breve, a degradação de RNA permanece uma potencial fonte de viés e pode afetar análises transcriptômicas. Avaliação rigorosa da integridade do RNA é, portanto, essencial para assegurar validade e reprodutibilidade, e será foco de trabalhos futuros.

## Conclusão

ZEB1-AS1 está marcadamente superexpresso em tecidos com HVE e em células AC16 estimuladas com ISO. Funcionalmente, ZEB1-AS1 promove a HC atuando como ceRNA para miR-186-5p a fim de modular a expressão de HDAC2. Esses achados definem um eixo ZEB1-AS1/miR-186-5p/HDAC2 na patogênese da HC e fornecem base mecanística para explorar estratégias terapêuticas direcionadas.
