# Supplementary material for: Knockdown do RNA Longo Não Codificante ZEB1-AS1 Acelera a Hipertrofia Cardíaca pela Via miR-186-5p/HDAC2
Source: Arq Bras Cardiol. 2025 Dec 18;122(11):e20240703. [Article in Portuguese] doi: 10.36660/abc.20240703 (PMC12978282; doi:10.36660/abc.20240703)
Supplement: Translation

# Translation: Knockdown of the Long Noncoding RNA ZEB1-AS1 Accelerates Cardiac Hypertrophy via the miR-186-5p/HDAC2 Pathway

**DOI:** 10.36660/abc.20240703i

**Authors:** Bingfeng Cao, Qingxia Liu, Xiaoyuan Zhang, Yufeng Wang

**Affiliations:** 1 China Department of Cardiology – Weihai Central Hospital, Shandong – China; 2 China Department of Outpatient – Weihai Central Hospital, Shandong – China

**Keywords:** Cardiomegaly, Long Noncoding RNA, Histone Deacetylase 2

## Abstract

**Background:**

The role of the long noncoding RNA ZEB1-AS1 in cardiac hypertrophy (CH) remains unclear.

**Objectives:**

To investigate the function of ZEB1-AS1 in the development and progression of CH as well as elucidate its underlying molecular mechanism.

**Methods:**

RNA expression levels were quantified by quantitative real-time PCR. Surface area of AC16 cells was assessed by immunofluorescence staining. Protein expression was evaluated by Western blotting. Interactions among RNAs were examined using luciferase reporter assays and RNA immunoprecipitation. Statistical significance was set at p < 0.05.

**Results:**

The expression of ZEB1-AS1 was upregulated in myocardial tissues and in isoproterenol (ISO)-stimulated AC16 cells. The knockdown of ZEB1-AS1 mitigated ISO-induced hypertrophic responses. Mechanistically, ZEB1-AS1 modulated histone deacetylase 2 (HDAC2) expression by acting as a molecular sponge for miR-186-5p. Consistently, the knockdown of ZEB1-AS1 reduced HDAC2 and decreased the expression of hypertrophic markers, including B-type natriuretic peptide, atrial natriuretic peptide, and β-myosin heavy chain, thereby restraining the progression of CH.

**Conclusions:**

ZEB1-AS1 is upregulated in myocardial tissues and ISO-stimulated AC16 cells. Our findings indicate the ZEB1-AS1/miR-186-5p/HDAC2 axis contributes to CH, providing a mechanistic basis and potential therapeutic target for clinical intervention.

## Introduction

Cardiac hypertrophy (CH) is characterized by enlargement of cardiomyocytes rather than an increase in their number. It primarily represents a compensatory response to biomechanical stress, intended to preserve cardiac output and maintain physiological homeostasis.^1,2^ CH is broadly classified as pathological or physiological. Pathological CH is commonly associated with cardiomyocyte apoptosis, myocardial fibrosis, disorganized cellular architecture, epigenetic alterations, and aberrant reactivation of fetal gene programs, including the markers B-type natriuretic peptide (BNP), atrial natriuretic peptide (ANP), and β-myosin heavy chain (β-MHC).^3-5^ Persistent pathological CH can lead to serious clinical outcomes, such as arrhythmia, maladaptive cardiac remodeling, heart failure (HF), myocardial infarction, and sudden cardiac death.^6,7^ Despite advances in therapy, outcomes — particularly for CH-related HF — remain suboptimal, with about 50% survival at 5 years after diagnosis.^8^ Elucidating the molecular mechanisms driving the development and progression of CH is therefore an urgent research priority.

Noncoding RNAs (ncRNAs) — including circular RNAs (circRNAs), long noncoding RNAs (lncRNAs), and microRNAs (miRNAs) — have attracted considerable attention for their roles in diverse regulatory processes, primarily through modulation of protein-coding RNA expression.^9-13^ LncRNAs, defined as noncoding transcripts ≥ 200 nucleotides that lack protein-coding capacity, have emerged as key regulators across a wide spectrum of pathological conditions.^14,15^ Numerous studies show lncRNAs contribute to the pathogenesis of diseases such as diabetic nephropathy,^16^ pulmonary hypertension,^17^ malignancies,^18^ and cardiovascular diseases.^19-21^ Growing evidence also implicates lncRNAs in the development and progression of CH. For example, silencing lncRNA TUG1 attenuates CH by modulating the miR-497/MEF2C pathway,^22^ ncRNA PVT1 promotes pathological CH by acting as a molecular sponge for miR-196b,^23^ and lncRNA NEAT1 accelerates CH via the miR-19a-3p/SMYD2 axis.^24^ The long noncoding RNA ZEB1-AS1 (lncRNA ZEB1-AS1), a recently identified lncRNA initially studied in cancer, has been implicated in multiple pathological processes;^25^ however, its role in CH remains largely undefined.

MiRNAs are a class of highly conserved, short ncRNAs (typically > 22 nucleotides [nt]) that repress target mRNA expression.^26^ miR-186-5p is a well-characterized miRNA involved in the regulation of several human diseases, including cardiovascular conditions.^27-29^ Nevertheless, its specific role in CH is not well understood. Histone deacetylase 2 (HDAC2), a member of the histone deacetylase family, has been implicated in various pathological states, including CH.^30-32^ Whether the miR-186-5p/HDAC2 regulatory axis participates in ZEB1-AS1–mediated CH progression remains to be determined.

This study aimed to investigate the role of ZEB1-AS1 in CH development and progression and delineates its underlying mechanism in order to identify a potential therapeutic target.

## Methods

### Human heart tissues specimens

We collected human cardiac tissue specimens from 20 patients with left ventricular hypertrophy (LVH) — heart sections obtained during heart transplantation — and from 10 normal donor hearts without a history of cardiac disease, at our hospital between March 2018 and September 2023. Table 1 summarizes clinical characteristics of patients and donors.

**Table 1:** – Primer sequences used for qRT-PCR

Gene	Forward (5′ → 3′)	Reverse (5′ → 3′)
ZEB1-AS1	5′-CCGTGGGCACTGCTGAAT-3′	5′-CTGCTGGCAAGCGGAACT-3′
miR-186-5p	5′-CAAAGAAUUCUCCUUUUGGGCU-3′	5′-CCCAAAAGGAGAAUUCUUUGUU-3′
HDAC2	5′-TGAGATTCCCAATGAGTTGCCA-3′	5′-TACTGACATCTGGTCAGACA-3′
ANP	5′-CAACGCAGA-CCTGATGGATTT-3′	5′-AGCCCCCGC-TTCTTCATTC-3′
BNP	5′-TGGAAACGTCCGGGTTACAG-3′	5′-CTGATCCGGTCCATCTTCCT-3′
β-MHC	5′-CCGAGTCCCAGGTCAACAA-3′	5′-CTTCACGGGCACCCTTGGA-3′
U6	5′-GCTTCGGCAGCACATATACTAAAAT-3′	5′-CGCTTCACGAATTTGCGTGTCAT-3′
GAPDH	5′-CCATCAACGACCCCTTCATT-3′	5′-GACCAGCTTCCCATTCTCAG-3′

ANP: atrial natriuretic peptide; BNP: B-type natriuretic peptide; β-MHC: beta-myosin heavy chain; GAPDH: glyceraldehyde-3-phosphate dehydrogenase; HDAC2: histone deacetylase 2; miR-186-5p: microRNA-186-5p; qRT-PCR: quantitative real-time polymerase chain reaction; U6: U6 small nuclear RNA; ZEB1-AS1: zinc finger E-box binding homeobox 1 antisense RNA 1.

Inclusion criteria for LVH were interventricular septal thickness at end-diastole and/or LV posterior wall depth ≥ 1.2 cm, with a LV ejection fraction > 40% on echocardiography. Exclusion criteria included systolic HF, malignancy, acute infection, BNP > 400 ng/L, Parkinson’s disease, idiopathic pulmonary fibrosis, shock, liver cirrhosis, or recent treatment with interferon or phenobarbital.^33^ Donor hearts were from individuals who died of noncardiac causes; tissues were collected within 0.5–6 h postmortem. All cardiac tissue specimens were immediately snap-frozen in liquid nitrogen (Delun, Shanghai, China) before RNA extraction.

The study was approved by the Institutional Ethics Committee of our hospital, and informed consent was obtained from all participants. Research procedures adhered to the principles of the 2024 revision of the World Medical Association Declaration of Helsinki.^34^

### Cell culture and transfection

Human cardiomyocyte cell line AC16 was obtained from the American Type Culture Collection (USA). Cells were cultured in Dulbecco’s Modified Eagle Medium (HyClone Laboratories, USA) supplemented with 20% fetal bovine serum (Gibco, USA) and maintained in a humidified incubator at 37 °C with 5% CO_2_. In order to establish an in vitro model of CH, AC16 cells were treated with 10 µmol/L isoproterenol (ISO; Sigma-Aldrich, USA) according to published protocols.^35^

Short hairpin RNA–negative control (sh-NC) and sh-ZEB1-AS1 constructs were obtained from GeneChem (Shanghai, China). miR-186-5p mimics, a miR-186-5p inhibitor, and their corresponding negative controls were purchased from RiboBio (Guangzhou, China). Cell transfections were performed with Lipofectamine 3000 (Sigma-Aldrich, USA) following the manufacturer’s instructions.

### Quantitative real-time PCR

Total RNA was extracted from human heart tissue specimens and AC16 cells using TRIzol reagent (Sigma-Aldrich, USA). cDNA synthesis and amplification were performed with a SYBR Green Master Mix kit (Takara, Japan) following the manufacturer’s instructions. GAPDH and U6 served as endogenous controls for normalization of lncRNA/mRNA and miRNA expression, respectively. Relative expression was calculated using the 2^Ct^ method. Table 2 lists primer sequences used for qRT-PCR.

**Table 2:** – Clinical characteristics of healthy donors and patients with LVH

Variable	Healthy (n = 10)	LVH (n = 20)	p-value
Age (years)	67 ± 8	68 ± 12	0.8141
Male/female (n/n)	4/6	11/9	0.4390
Smoking, n (%)	4 (40%)	11 (55%)	0.4390
DM, n (%)	3 (30%)	12 (60%)	0.1210
Hypertension, n (%)	4 (40%)	18 (90%)	0.0040
ACS, n (%)	2 (20%)	14 (70%)	0.0100
AF, n (%)	0 (0%)	5 (25%)	0.0830
Fasting glucose (mmol/L)	5.51 ± 0.54	6.23 ± 1.85	0.2417
SBP (mmHg)	115.25 ± 18.25	159.91 ± 28.24	0.0001
DBP (mmHg)	73.52 ± 12.25	95.41 ± 15.36	0.0005
HDL-C (mmol/L)	1.36 ± 0.11	1.15 ± 0.29	0.0365
LDL-C (mmol/L)	2.61 ± 0.56	3.05 ± 0.81	0.1354
BNP (ng/L)	129.61 ± 74.12	158.36 ± 95.21	0.4112
EF (%)	78.55 ± 5.81	58.25 ± 6.25	0.0001
LV diameter (cm)	4.25 ± 0.45	4.38 ± 0.66	0.5807
LVIDd (cm)	4.55 ± 0.50	4.99 ± 0.91	0.1673
IVSd (cm)	0.98 ± 0.08	1.24 ± 0.13	0.0001
PWd (cm)	0.95 ± 0.12	1.29 ± 0.12	0.0001
Relative ZEB1-AS1 (mean ± SD)	0.895 ± 0.394	2.194 ± 0.633	0.0001

ACS: acute coronary syndrome; AF: atrial fibrillation; BNP: B-type natriuretic peptide; DBP: diastolic blood pressure; DM: diabetes mellitus; HDL-C: high-density lipoprotein cholesterol; IVSd: interventricular septum thickness at end-diastole; LDL-C: low-density lipoprotein cholesterol; LV: left ventricle; LVEF: LV ejection fraction; LVIDd: LV internal dimension at end-diastole; LVH: LV hypertrophy; PWd: LV posterior wall thickness at end-diastole; SBP: systolic blood pressure.

### Western blotting

Protein extraction from AC16 cells was performed as described previously.^36^ Briefly, cells were lysed in radioimmunoprecipitation assay buffer and proteins were denatured by boiling. Equal amounts of protein were resolved on 10% sodium dodecyl sulfate-polyacrylamide gel electrophoresis and transferred to polyvinylidene difluoride membranes (Millipore, USA). Membranes were blocked with 1% bovine serum albumin (BSA) for 1 h at room temperature and washed with phosphate-buffered saline (PBS). Blots were incubated overnight at 4 °C with primary antibodies against ANP (1:2000, Abcam, USA), BNP (1:2000, CST, USA), β-MHC (1:2000, CST, USA), and GAPDH (1:2000, Abcam, USA). After washing, membranes were incubated with secondary antibody (1:10,000, Jackson, USA) for 2 h at room temperature. Bands were visualized using enhanced chemiluminescence (Amersham, UK) and quantified in ImageJ (National Institutes of Health, USA).

### Immunofluorescent staining

AC16 cells were fixed with 4% paraformaldehyde at 37 °C for 30 min, washed three times with PBS, and permeabilized with 0.1% Triton X-100 for 15 min. Cells were blocked for 30 min in 10% normal goat serum with 1% BSA (Sigma-Aldrich). They were then incubated overnight at 4 °C in the dark with an anti–β-actin primary antibody (Abcam, USA) diluted in blocking buffer. After three PBS washes, cells were incubated with the appropriate secondary antibody for 2 h at 37 °C, counterstained with DAPI, and imaged on a fluorescence microscope (Carl Zeiss). Cell surface area was quantified using ImageJ.

### Dual-luciferase reporter assay

Binding sequences for miR-186-5p within ZEB1-AS1 and the HDAC2 3′-UTR were cloned into pGL3-Basic vectors (LMAI Bio, Shanghai, China). AC16 cells were co-transfected with either negative-control mimic or miR-186-5p mimic together with the wild-type (WT) or mutant (MUT) reporter constructs. After transfection, cells were harvested and luciferase activity was measured using the Dual-Luciferase Reporter Assay System (Promega, USA).

### RNA immunoprecipitation

RNA immunoprecipitation (RIP) was performed with the RNA-Binding Protein Immunoprecipitation Kit (Millipore, USA) to assess interactions among ZEB1-AS1, miR-186-5p, and HDAC2, following the manufacturer’s instructions. Briefly, cells were lysed in RIP lysis buffer and incubated with magnetic beads conjugated to anti-Argonaute-2 (Ago2; Abcam, USA) or control rabbit IgG (Abcam, USA). After three PBS washes, RNA co-precipitated with Ago2 or IgG was purified and analyzed by qRT-PCR.

### Data analysis

Analyses were performed in GraphPad Prism version 7.0.0 for Windows, GraphPad Software, Boston, Massachusetts USA, www.graphpad.com. Data are presented as mean ± standard deviation (SD). Two-group comparisons used an unpaired two-tailed Student *t* test; multiple-group differences were assessed by one-way ANOVA with Bonferroni post hoc testing. Associations between ZEB1-AS1 expression and clinical variables were evaluated with the chi-square test. Normality and homoscedasticity were assessed by the Shapiro-Wilk and Levene tests, respectively; p > 0.05 indicates the assumptions were met. Statistical significance was set at p < 0.05.

## Results

### ZEB1-AS1 in patients with LV and in ISO-induced CH in AC16 cells

We first quantified ZEB1-AS1 in heart tissues from 20 patients with LVH and 10 normal donor hearts by qRT-PCR. ZEB1-AS1 was significantly upregulated in LVH tissues versus controls (Figure 1A).

To model CH in vitro, AC16 cells were treated with ISO (10 µM). ISO increased cell surface area at 24 h and reached a maximum at 48 h relative to untreated controls (Figure 1B). In parallel, ISO elicited time-dependent increases in the mRNA and protein levels of hypertrophic markers — namely BNP, ANP, and β-MHC — compared with controls (Figures 1C and 1D), confirming successful establishment of the CH model.

Notably, ZEB1-AS1 expression was also significantly increased in ISO-treated AC16 cells versus controls (Figure 1E), suggesting a role for ZEB1-AS1 in CH progression.

**Figure 1:** – Upregulation of ZEB1-AS1 in LVH tissues and ISO-stimulated AC16 cells. A) ZEB1-AS1 expression in LVH hearts (n = 20) versus normal donors (n = 10) by qRT-PCR. B) AC16 cell surface area after PBS or ISO treatment assessed by immunofluorescence. C) mRNA levels of BNP, ANP, and β-MHC in AC16 cells after PBS or ISO by qRT-PCR. D) Protein levels of BNP, ANP, and β-MHC in AC16 cells after PBS or ISO by Western blot. E) ZEB1-AS1 expression in AC16 cells after PBS or ISO by qRT-PCR. All experiment was performed in triplicate. *p < 0.05, **p < 0.01. ANP: atrial natriuretic peptide; BNP: B-type natriuretic peptide; β-MHC: beta-myosin heavy chain; CH: cardiac hypertrophy; ISO: isoproterenol; LVH: left ventricular hypertrophy; PBS: phosphate-buffered saline; qRT-PCR: quantitative real-time polymerase chain reaction.

### ZEB1-AS1 deficiency mitigates ISO-induced CH in AC16 cells

To assess the role of ZEB1-AS1 in ISO-induced hypertrophy, AC16 cells were transfected with sh-ZEB1-AS1 or sh-NC. ZEB1-AS1 expression was significantly reduced in sh-ZEB1-AS1 cells versus sh-NC, confirming efficient knockdown (Figure 2A). ISO treatment markedly increased AC16 cell surface area, consistent with hypertrophic changes, whereas this enlargement was significantly attenuated in sh-ZEB1-AS1–transfected cells (Figure 2B). Likewise, ISO upregulated the mRNA and protein levels of hypertrophic markers (namely, BNP, ANP, and β-MHC); this induction was substantially blunted by ZEB1-AS1 knockdown (Figure 2C,D). These data indicate ZEB1-AS1 downregulation negatively regulates ISO-induced CH.

**Figure 2:** – ZEB1-AS1 deficiency mitigates ISO-induced CH in AC16 cells. A) ZEB1-AS1 expression in AC16 cells transfected with sh-NC or sh-ZEB1-AS1 by qRT-PCR; B) AC16 cell surface area after ISO stimulation with sh-NC or sh-ZEB1-AS1 assessed by immunofluorescence; C) mRNA levels of BNP, ANP, and β-MHC in ISO-stimulated AC16 cells with sh-NC or sh-ZEB1-AS1 by qRT-PCR; D) protein levels of BNP, ANP, and β-MHC in ISO-stimulated AC16 cells with sh-NC or sh-ZEB1-AS1 by Western blot. All experiments were performed in triplicate. *p < 0.05, **p < 0.01. ANP: atrial natriuretic peptide; BNP: B-type natriuretic peptide; β-MHC: beta-myosin heavy chain; CH: cardiac hypertrophy; ISO: isoproterenol; qRT-PCR: quantitative real-time polymerase chain reaction; sh-NC: short hairpin RNA–negative control; sh-ZEB1-AS1: ZEB1-AS1-targeting shRNA.

### ZEB1-AS1 regulates HDAC2 expression by sequestering miR-186-5p

We used starBase 2.0 to predict miRNAs that interact with ZEB1-AS1 and identified miR-186-5p as a candidate (Figure 3A). A dual-luciferase reporter assay showed that co-transfection of miR-186-5p mimic with the ZEB1-AS1 wild-type (ZEB1-AS1-WT) construct significantly reduced luciferase activity in AC16 cells, whereas the mutant construct (ZEB1-AS1-MUT) had no effect (Figure 3B), confirming specific binding. Using TargetScan, we next identified HDAC2 as a putative downstream target of miR-186-5p (Figure 3C). This interaction was validated by luciferase assay: the miR-186-5p mimic decreased activity of the HDAC2-WT reporter but not the HDAC2-MUT reporter (Figure 3D). RIP further demonstrated enrichment of ZEB1-AS1, miR-186-5p, and HDAC2 in Ago2 immunoprecipitates from AC16 cell lysates (Figure 3E), indicating co-association within the RNA-induced silencing complex.

In myocardial tissues from 20 patients with LVH versus 10 normal donor hearts, qRT-PCR revealed lower miR-186-5p and higher HDAC2 expression (Figure 3F,G). Together, these data support a ZEB1-AS1/miR-186-5p/HDAC2 axis in CH, whereby ZEB1-AS1 modulates HDAC2 by sponging miR-186-5p.

**Figure 3:** – ZEB1-AS1 regulates HDAC2 by sequestering miR-186-5p. A) Predicted miR-186-5p binding site in ZEB1-AS1 (WT) and corresponding mutant (MUT) construct. B) Dual-luciferase assay showing reduced reporter activity for ZEB1-AS1-WT, but not ZEB1-AS1-MUT, in the presence of miR-186-5p mimic. C) Predicted miR-186-5p binding site in the HDAC2 3′-UTR (WT) and MUT construct. D) Dual-luciferase assay showing decreased activity for HDAC2-WT, but not HDAC2-MUT, with miR-186-5p mimic. E) RIP demonstrating enrichment of ZEB1-AS1, miR-186-5p, and HDAC2 in Ago2 immunoprecipitates. F-G) miR-186-5p and HDAC2 levels in LVH hearts (n = 20) versus normal donors (n = 10) by qRT-PCR. All experiments were performed in triplicate. *p < 0.05, **p < 0.01. Ago2: Argonaute-2; CMV: cytomegalovirus; HDAC2 histone deacetylase 2; LVH: left ventricular hypertrophy; miR-NC: negative-control mimic; MUT: mutant; qRT-PCR: quantitative real-time polymerase chain reaction; RIP: RNA immunoprecipitation; UTR: untranslated region; WT: wild type.

### ZEB1-AS1 promotes CH by regulating the miR-186-5p/HDAC2 pathway

To test whether ZEB1-AS1 drives CH via the miR-186-5p/HDAC2 axis, we performed rescue experiments. HDAC2 mRNA was measured in ISO-stimulated AC16 cells transfected with sh-NC, sh-ZEB1-AS1, or co-transfected with sh-ZEB1-AS1 plus a miR-186-5p inhibitor. ZEB1-AS1 knockdown significantly reduced HDAC2 mRNA, and this reduction was reversed by miR-186-5p inhibition (Figure 4A). Likewise, the ISO-induced increase in cell surface area was attenuated by sh-ZEB1-AS1, whereas concurrent inhibition of miR-186-5p abrogated this effect (Figure 4B). In parallel, ZEB1-AS1 knockdown decreased the ISO-induced mRNA and protein expression of hypertrophic markers (namely, BNP, ANP, and β-MHC), and these suppressive effects were reversed by miR-186-5p inhibition (Figures 4C and 4D). Collectively, these results indicate ZEB1-AS1 promotes CH by regulating the miR-186-5p/HDAC2 signaling pathway.

**Figure 4:** – ZEB1-AS1 promotes CH by regulating the miR-186-5p/HDAC2 pathway. A) HDAC2 mRNA in ISO-stimulated AC16 cells transfected with the indicated constructs measured by qRT-PCR; B) AC16 cell surface area after ISO stimulation and transfection with the indicated constructs assessed by immunofluorescence; C) mRNA levels of BNP, ANP, and β-MHC in ISO-stimulated AC16 cells after transfection measured by qRT-PCR; D) protein levels of BNP, ANP, and β-MHC in ISO-stimulated AC16 cells after transfection measured by Western blot. All experiments were performed in triplicate. *p < 0.05, **p < 0.01. ANP: atrial natriuretic peptide; BNP: B-type natriuretic peptide; β-MHC: beta-myosin heavy chain; CH: cardiac hypertrophy; HDAC2: histone deacetylase 2; ISO: isoproterenol; qRT-PCR: quantitative real-time polymerase chain reaction; sh-NC: short hairpin RNA–negative control; sh-ZEB1-AS1: zinc finger E-box binding homeobox 1 antisense RNA 1-targeting short hairpin RNA; miR-186-5p: microRNA-186-5p.

## Discussion

In this study, we show ZEB1-AS1 is significantly upregulated in CH and its silencing attenuates ISO-induced hypertrophic responses in AC16 cells. Mechanistically, dual-luciferase and RIP assays confirmed miR-186-5p is a direct target of ZEB1-AS1 and HDAC2 is targeted by miR-186-5p, establishing a regulatory axis that contributes to CH progression.

LncRNAs were once considered nonfunctional transcriptional byproducts,^37^ but accumulating evidence indicates they regulate diverse biological processes through interactions with miRNAs, transcription factors, genomic DNA, and RNA-binding proteins. LncRNAs are increasingly recognized in cardiac development and in the pathogenesis of cardiovascular disease. For example, lncRNA MIR217HG exacerbates pressure-overload–induced CH via the miR-138/THBS1 pathway,^38^ ADAMTS9-AS1/miR-185-5p/KAT7 acts as a suppressive ceRNA network in hypertrophic obstructive cardiomyopathy,^39^ and the knockdown of DANCR mitigates HF by attenuating CH and fibrosis through the miR-758-3p/PRG4/Smad axis.^35^ ZEB1-AS1, initially characterized in cancer, has also been implicated across malignancies,^25,40,41^ but its role in CH remained unclear. Here, we identify ZEB1-AS1 as a pro-hypertrophic lncRNA: it is increased in LVH tissues and in ISO-treated AC16 cells, and its knockdown blunts ISO-induced hypertrophy, indicating a pathogenic role in CH development and progression.

LncRNAs frequently act within ceRNA networks to regulate gene expression post-transcriptionally by sequestering miRNAs and thereby modulating their availability to target mRNAs.^42,43^ The importance of ceRNA circuitry in CH has been substantiated in a number of studies.^44-46^ Consistently, we show ZEB1-AS1 functions as a sponge for miR-186-5p — a miRNA reported to be downregulated in cardiac tissue in diabetic cardiomyopathy^47^ — thus relieving repression of HDAC2 and promoting ISO-induced hypertrophy. To our knowledge, this is the first report implicating miR-186-5p in CH, highlighting its potential as a therapeutic entry point.

HDACs are key contributors to cardiovascular pathology.^30-32,48-50^ Prior work have shown that HDAC inhibition attenuates CH induced by pressure overload or ISO infusion,^51,52^ in line with our ISO-based in vitro model. Among HDACs, HDAC2 has received particular attention; phosphorylation at serine 394 is a critical regulatory event in CH progression.^53^ For example, the anti-hypertrophic effect of plantamajoside in ISO-induced CH is closely associated with HDAC2,^32^ and EYA4-au1 promotes cardiomyocyte hypertrophy by recruiting Med11 to upregulate EYA4, leading to p27^Kip^^1^ downregulation and activation of the CK2α/HDAC2 axis.^54^ Despite HDAC2’s established role, its upstream regulation via lncRNA–miRNA–mRNA networks has been less clear. Our data indicate HDAC2 is a direct target of miR-186-5p and ZEB1-AS1 promotes CH by modulating the miR-186-5p/HDAC2 axis.

### Limitations of study

This study has some limitations. Because lncRNAs often interact with multiple miRNAs and individual miRNAs typically regulate numerous targets, ZEB1-AS1 may influence additional pathways relevant to CH that were not examined here. These alternative mechanisms warrant further investigation. In addition, although the postmortem interval for autopsy-derived tissues was brief, RNA degradation remains a potential source of bias and could affect transcriptomic analyses. Rigorous assessment of RNA integrity is therefore essential to ensure validity and reproducibility, and will be a focus of future work.

## Conclusion

ZEB1-AS1 is markedly upregulated in LVH tissues and in ISO-stimulated AC16 cells. Functionally, ZEB1-AS1 promotes CH by acting as a ceRNA for miR-186-5p to modulate HDAC2 expression. These findings define a ZEB1-AS1/miR-186-5p/HDAC2 axis in CH pathogenesis and provide a mechanistic basis for exploring targeted therapeutic strategies.
